# Twist1 confers multidrug resistance in colon cancer through upregulation of ATP-binding cassette transporters

**DOI:** 10.18632/oncotarget.17548

**Published:** 2017-05-02

**Authors:** Yan-Rong Liu, Lan Liang, Jian Min Zhao, Yang Zhang, Min Zhang, Wei-Long Zhong, Qiang Zhang, Jun-Jie Wei, Meng Li, Jie Yuan, Shuang Chen, Shu-Min Zong, Hui-Juan Liu, Jing Meng, Yuan Qin, Bo Sun, Lan Yang, Hong-Gang Zhou, Tao Sun, Cheng Yang

**Affiliations:** ^1^ Tianjin Key Laboratory of Molecular Drug Research, Tianjin International Joint Academy of Biomedicine, Tianjin, China; ^2^ State Key Laboratory of Medicinal Chemical Biology and College of Pharmacy, Nankai University, Tianjin, China; ^3^ Tianjin GoalGen Biotechnology Co., Ltd., Tianjin, China; ^4^ Pathology Department, Shun Yi District Hospital, Beijing, China; ^5^ Department of Anesthesiology, Tianjin 4th Center Hospital, Tianjin, China

**Keywords:** Twist1, multidrug resistance, colon cancer, ABCB1, ABCC1

## Abstract

Multidrug resistance is a major problem in colon cancer treatment. However, its molecular mechanisms remain unclear. Recently, the epithelial-mesenchymal transition (EMT) in anticancer drug resistance has attracted increasing attention. This study investigated whether vincristine treatment induces EMT and promotes multidrug resistance in colon cancer. The result showed that vincristine treatment increases the expression of several ATP-binding cassette transporters in invasive human colon adenocarcinoma cell line (HCT-8). Vincristine-resistant HCT-8 cells (HCT-8/V) acquire a mesenchymal phenotype, and thus its migratory and invasive ability are increased both *in vitro* and *in vivo*. The master transcriptional factors of EMT, especially Twist1, were significantly increased in the HCT-8/V cell line. Moreover, the ectopic expression of Twist1 increased the chemoresistance of HCT-8 cells to vincristine and increased the expression levels and promoter activities of ABCB1 and ABCC1. Furthermore, Twist1 silencing reverses the EMT phenotype, enhances the chemosensitivity of HCT-8/ V cells to anticancer agents *in vitro* and *in vivo*, and downregulates the expression of ABCB1 and ABCC1. Twist1-mediated promotion of ABCB1 and ABCC1 expression levels plays an important role in the drug resistance of colon cancer cells.

## INTRODUCTION

Chemotherapy medications, such as vincristine, remain a vital treatment for advanced colon cancer [1]. However, the development of treatments is becoming increasingly complex because of the resistance to multiple chemotherapeutic agents. Multidrug resistance (MDR) is a phenomenon wherein tumor cells exhibit cross-resistance against structurally unrelated cytotoxic agents [2]. The underlying mechanisms of this phenomenon in colon cancer are poorly understood.

ABC transporter superfamily is the largest transportome in the human genome [3]. Increased ABC transporter expression has been related with aggressive cancers [4], which also tend to be more chemoresistant. Epithelial-mesenchymal transition (EMT) is a vital stage for malignant tumor progression; emerging evidence has shown that EMT promoted MDR. Transcription regulators of EMT, such as Snail1 and Twist1, have been described to participate in the chemoresistance regulation in lung, breast, and cervical cancers [5–7]. The present study aims to determine whether EMT participates in MDR of colon cancer.

The results showed that vincristine resistance induces MDR and promotes the proliferation and metastasis of colon cancer cells. In addition, vincristine resistance-induced EMT played vital roles in MDR by modulating ABCB1 and ABCC1 expression levels. Moreover, downregulation of Twist1 expression reversed the EMT; reduced the proliferating and metastatic ability and enhanced the chemosensitivity of the vincristine resistant cells. Thus, Twist1 is a promising therapeutic target against colon cancer in vincristine-based chemotherapy.

## RESULTS

### Heterogeneous expression of ABC transporters in different colon cancer cells

**Figure 1:**
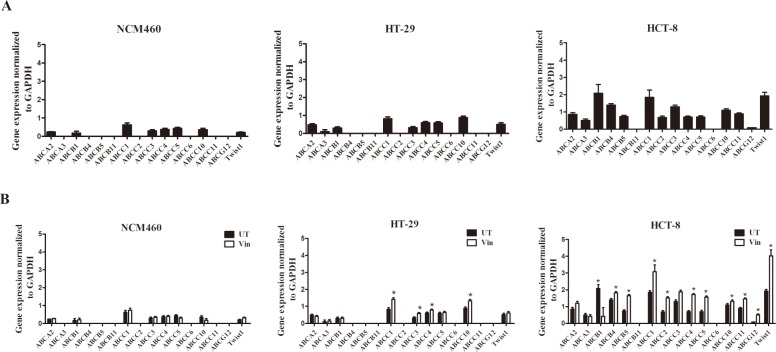
Heterogeneous expression of ABC transporters and Twist1 in different colon cancer cells **(A)** Basal relative mRNA expression of ABC transporters and Twist1 in immortalized NCM460, low-invasive (HT-29) and high-invasive cell types (HCT-8). **(B)** Vincristine treatment upregulates ABC transporter and Twist1 expression in invasive HCT-8 cells.

To assess the basal expression levels of the ABC transporters, which are implicated in drug resistance, we performed a detailed RT-PCR-based analysis in three human colon cell lines comprising immortalized, low-invasive, and high-invasive cell types. We found a large heterogeneity among the expression levels of the ABC transporters. The high-invasive colon cancer cell line HCT-8 had higher expression levels of ABC transporters compared with the low-invasive HT-29 cells and normal colon cell line NCM460 (Figure 1A), suggesting that the invasive potential of the tumor cells may increase the expression levels of the ABC transporters.

Moreover, to test the effects of the vincristine treatment on ABC transporter expression in colon cancer cells mentioned above, we exposed the cells to 0.1 μM of vincristine for two weeks. The results showed that vincristine treatment upregulated ABC transporter expression only in the invasive colon cancer cells (Figure 1B), implying a close correlation between the invasive phenotype and ABC transporter-mediated drug resistance in colon cancer cells.

The expression levels of Twist1, the key EMT-promoting transcription factors, were also detected in these cells as EMT has been described to participate in the chemoresistance regulation recently [5–7]. The results showed that the high-invasive colon cancer cell line HCT-8 had higher expression levels of Twist1 compared with the low-invasive HT-29 cells and normal colon cell line NCM460 and vincristine treatment upregulated Twist1 expression only in the invasive colon cancer cells (Figure 1A–1B).

### MDR of colon carcinoma led to changes in cell functions and proteins consistent with EMT

**Figure 2:**
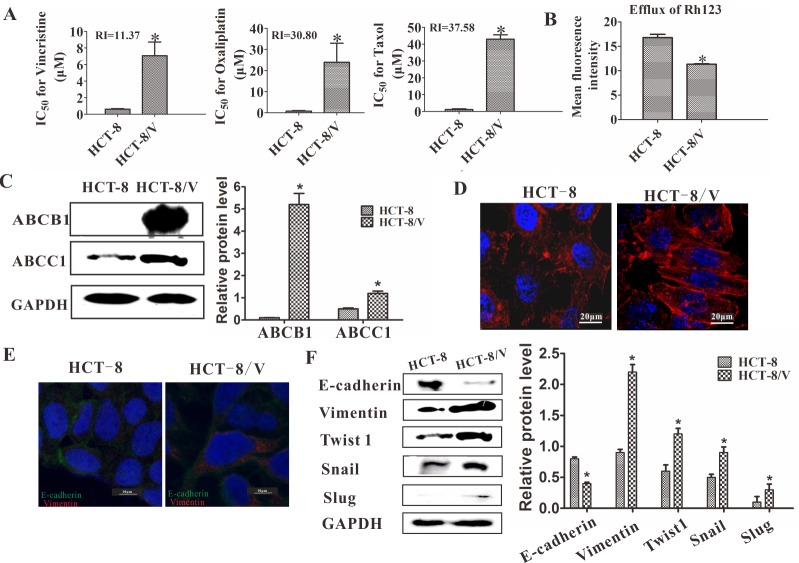
Effects of chemoresistance on the drug transporters and EMT **(A)** IC_50_ values of HCT-8/V and parental HCT-8 cells for vincristine, taxol, and cisplatin; RI represents the resistance index. **(B)** Rh123 efflux was measured using flow cytometry. **(C)** Western blot analysis of ABCB1 and ABCC1; GAPDH was used as a loading control. **(D)** Increased actin microfilament was observed in the HCT-8/V cells by cytoskeleton staining. **(E)** Immunofluorescence staining of epithelial and mesenchymal markers in HCT-8 and HCT-8/V cells. Cells were double-stained for vimentin (red) and E-cadherin (green). Nuclei were counterstained blue with DAPI. **(F)** Expression of EMT markers and transcription factors was determined by Western blot. GAPDH was used as a loading control. Scale bars represent 10 μm in **D** and **E**. Data from three independent experiments were graphed as the mean ± SD. *P < 0.05.

The HCT-8/V and HCT-8 cells were exposed to increasing concentrations of vincristine to detect their drug sensitivity. The IC_50_ values of vincristine in the HCT-8 and HCT-8/V cells were 0.622 and 7.07 μM, respectively. In addition, the HCT-8/V cells were treated with taxol and oxaliplatin to determine the drug sensitivity of other drugs. Taxol and oxaliplatin are chemotherapy drugs that are structurally and functionally unrelated to vincristine. The HCT-8/V cells were cross-resistant to these drugs and had RI values of 30.80 and 37.58, respectively (Figure 2A). Thus, HCT-8/V is a multidrug-resistant cell. The function of the drug transporter was also evaluated by analyzing the efflux of Rh123 through flow cytometry. As shown in Figure 2B, the fluorescence intensity of the HCT-8/V cells was significantly lower than that of the HCT-8 cells. Therefore, the efflux of the drug was higher in HCT-8/V cells that that of the HCT-8 cells. These results indicated that the sensitivity to chemotherapeutic drugs decreased in the former. Meanwhile, ABCB1 and ABCC1 were detected by Western blot analysis. As shown in Figure 2C, the expression levels of ABCB1 and ABCC1 in the HCT-8/V cell line were significantly higher than those in the parental cell line. Moreover, compared with HCT-8 cell line, HCT-8/V cell line displayed mesenchymal phenotype, such as the decreased cell–cell contacts and increased pseudopodia formation. Furthermore, the cytoskeleton staining results indicated that the actin microfilaments of the HCT-8/V cells were longer than those of the parental HCT-8 cells (Figure 2D). All these characteristics are similar to the typical cellular structures of EMT. The protein levels of the EMT markers in the HCT-8 cell lines were detected by immunofluorescence and Western blot analysis to further verify whether vincristine resistance could induce EMT in colon carcinoma. A significantly decreased expression level of E-cadherin and increased expression of vimentin were observed in the HCT-8/V cells (Figure 2E–2F). The expression levels of some well-known EMT regulators, such as Twist1, Snail, and Slug were analyzed by Western blot analysis to determine whether the transcription factors of EMT contributed to the vincristine-induced EMT. As shown in Figure 2F, the expression levels of Twist1, Snail, and Slug in the HCT-8/V cell line were comparably higher than those in the HCT-8 parental cells. Thus, EMT transcription factors may perform essential roles in the process of vincristine resistance.

### TGF-β-induced EMT leads to MDR of colon carcinoma

**Figure 3:**
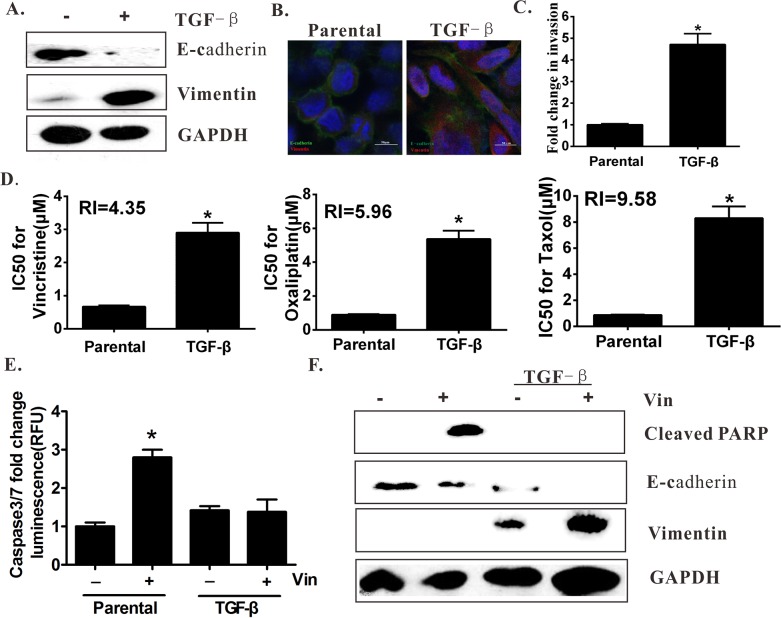
EMT induction by TGF-β is resistant to vincristine **(A)** Western blot analysis showed that TGF-β induction resulted in EMT and exhibited a loss of E-cadherin and a gain in vimentin expression. **(B)** EMT acquisition by TGF-β induction was characterized by immunofluorescence. **(C)** TGF-β-treated cells increased the invasion capacity in the transwell assays. **(D)** The drug sensitivity of the TGF-β-treated cells decreased compared with that of the parental cells. **(E)** TGF-β-treated cells failed to exhibit the apoptotic characteristics of caspase-3/7 activation after vincristine exposure. **(F)** Western blot analysis showed the effect of vincristine (Vin: 80 nM) on PARP cleavage.

TGF-β was used to induce EMT to demonstrate the EMT-mediated induction of MDR and examine whether the subsequent MDR can be observed. The results showed that exposure of the HCT-8 cells to TGF-β for 96 hours resulted in EMT, as indicated by the increased vimentin and decreased E-cadherin expression (Figure 3A–3B). In addition, the TGF-β-treated cells exhibited an increased invasive ability as determined by the transwell assays, and thus the mesenchymal phenotype was confirmed (Figure 3C). Moreover, the drug sensitivity of the TGF-β-treated cells was measured by exposing them to different concentrations of vincristine, taxol, and oxaliplatin. The results showed that the TGF-β-treated HCT-8 cells became more resistant to the drugs compared with the parental cells (Figure 3D). In the TGF-β-treated cells, vincristine failed to induce the expression or activation of apoptosis-related proteins, such as caspase-3/7 and PARP (Figure 3E–3F). Moreover, the blockade of TGF-β by the neutralizing antibody also diminished the drug resistance effects of rhTGF-β in colon cells (Supplementary Figure 1).

### Twist1 plays a vital role in MDR of colon carcinoma

**Figure 4:**
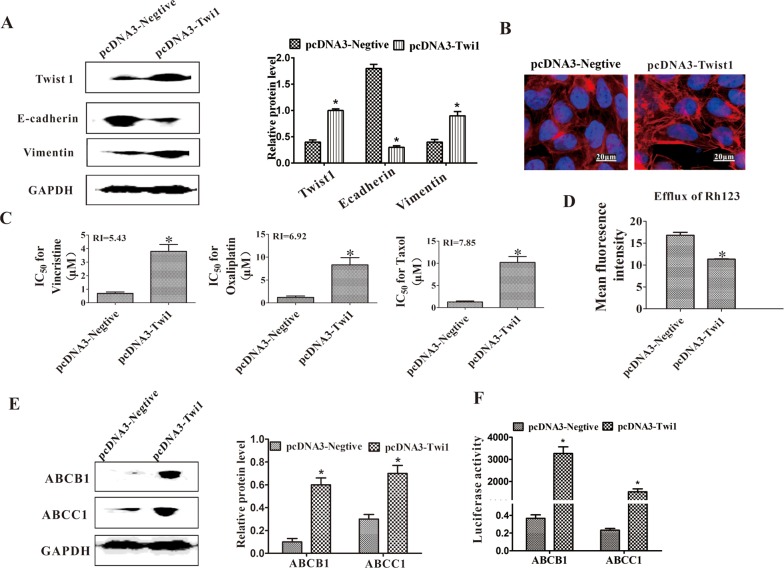
Ectopic expression of Twist1 induced EMT and MDR **(A)** Expression levels of Twist1, E-cadherin, and vimentin were determined through Western blot. GAPDH was used as a loading control. *P < 0.05. **(B)** Increased actin microfilament was observed in the HCT-8-T cells through cytoskeleton staining. **(C)** IC_50_ values of HCT-8-C and HCT-8-T cells for vincristine, taxol, and cisplatin. RI represents the resistance index. **(D)** Efflux of Rh123 was measured using flow cytometry. **(E)** Western blot analysis of ABCB1 and ABCC1. GAPDH was used as a loading control. **(F)** Overexpressing Twist1 increased ABCB1 and ABCC1 promoter activities in the luciferase reporter assays. Scale bars represent 50 μm. Data from three independent experiments are graphed as the mean ± SD. *P < 0.05.

Twist1 was overexpressed in the parental HCT-8 cells to verify the role of Twist1 in the MDR of colon carcinoma. The EMT was induced as indicated by the decreased expression of E-cadherin and increased expression of vimentin (Figure 4A), as well as the long and rich microfilaments (Figure 4B). The HCT-8 cells overexpressed with Twist1 were then exposed to different concentrations of vincristine, taxol, and oxaliplatin to test their drug sensitivity. After the ectopic expression of Twist1, the IC_50_ value of the HCT-8 cells increased (Figure 4C). The efflux of Rh123 was increased in Twist1-transfected HCT-8 cells (Figure 4D). As indicated by Western blot analysis results, the ABCB1 and ABCC1 expression levels were upregulated when transfected with Twist1 (Figure 4E). We confirmed the roles of Twist1 in the transcriptional activation of ABCB1 and ABCC1 by using luciferase reporter assays. The overexpression of Twist1 considerably increased ABCB1 and ABCC1 reporter activity, suggesting that Twist1 increased the transcriptional activation of ABCB1 and ABCC1 (Figure 4F). Moreover, Chip-PCR was used to furtherly confirm the role of Twist1 in upregulating the expression of ABCB1 by directly binding to their promoters (Supplementary Figure 2). Microarray assays were also performed to evaluate the drug resistance-related genes upregulated or downregulated by Twist1. It revealed that 36 differential genes related to drug resistance were found after Twist1 upregulation. Among these differential genes, 15 MDR promoting genes were upregulated. For example, Twist1 increased the expression levels of ABCA1 three times, which acts as a drug resistance transporter involved in many tumors. These assays further validated the important role of Twist1 in MDR (Supplementary Figure 3).

### Expression of Twist1, ABCB1 and response to vincristine chemotherapy

Advanced colorectal carcinoma specimens from patients who were primarily treated with surgery and postoperative vincristine-based chemotherapy were collected. Response to treatment was compared between high and low expression of Twist1 and ABCB1. The results showed that patients with both higher Twist1 and ABCB1 expression tumors had poorer response to chemotherapy (Supplementary Figure 4, Supplementary Table 4), suggesting that high expression of Twist1 and ABCB1 is predictive of resistance to vincristine-based chemotherapy. More cases will be collected in the future to further confirm our findings.

### Twist1 silencing reversed the EMT of HCT-8/V cells

**Figure 5:**
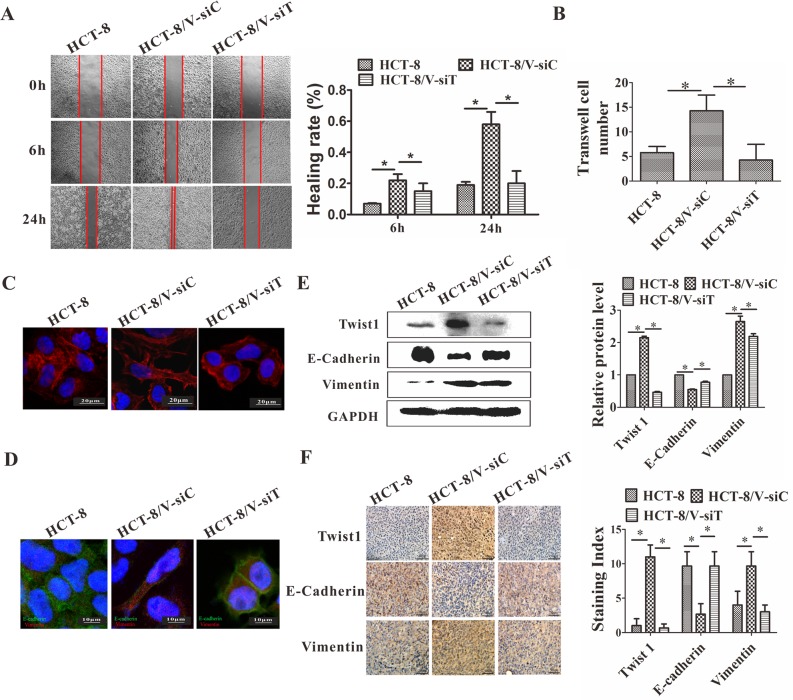
siRNA-mediated downregulation of Twist1 reversed EMT **(A)** Healing rate was assayed at 6 and 24 h. The left panel shows representative images; the right panel shows quantitative analysis. **(B)** Number of cells invaded through basement member. **(C)** Twist1 silencing significantly decreased the number of actin microfilaments. **(D)** Immunofluorescence staining of E-cadherin and vimentin in the HCT-8/V cells following the downregulation of Twist1. Green corresponds to E-cadherin; red corresponds to vimentin. A blue signal represents nuclear DNA staining by DAPI. **(E)** Western blot analysis of E-cadherin and vimentin was performed on the HCT-8/V cells following the inhibition of Twist1. GAPDH was used as a loading control. The right panel shows the quantitative analysis of the relative protein levels. **(F)** IHC staining for E-cadherin and vimentin in the HCT-8 transplanted tumors. Representative images are shown in the left panel; the staining index is shown in the right panel. Scale bars represent 50 μm in **D** and **F** and 10 μm in **C**. Data from the three independent experiments are graphed as mean ± SD. *P < 0.05.

Twist 1 was silenced by transfection of Twist 1 siRNA to determine whether Twist 1 is necessary for vincristine-induced EMT. As shown in Figure 5A–5C, the Twist1 silencing decreased the migration and invasion ability, as determined by the wound healing and transwell assays. A decreased number of microfilaments were observed after Twist1 silencing. The downregulation of Twist1 reversed the EMT phenotype such that the E-cadherin expression increased and Vimentin expression decreased, as determined by Western blot analysis, immunofluorescence, and IHC staining (Figure 5D–5F). The silencing of Twist1 causes the reversal of EMT.

### Twist1 silencing reversed MDR of colon cells *in vitro*

**Figure 6:**
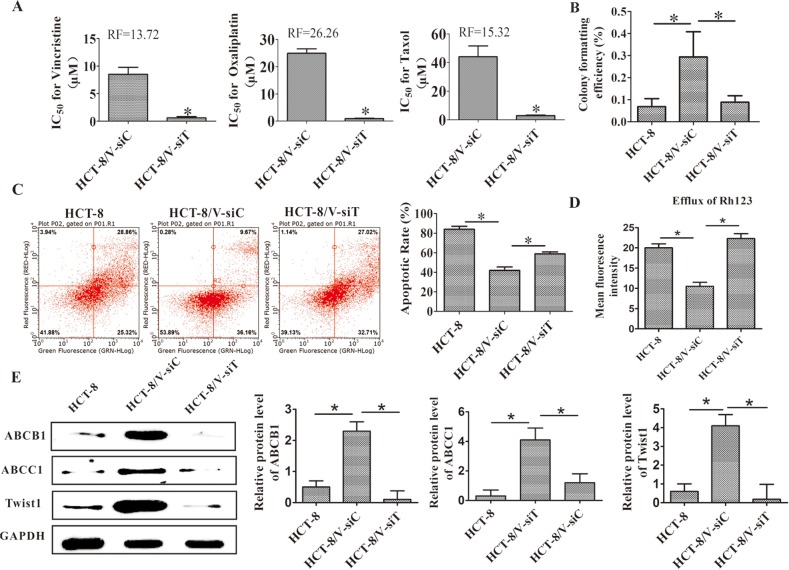
Downregulation of Twist1 reversed MDR *in vitro* **(A)** IC_50_ values for vincristine, taxol, and cisplatin. **(B)** Proliferating abilities of the HCT-8/V and HCT-8 cells were determined by colony formation assay. **(C)** Analysis on cell apoptosis by flow cytometry; the quantitative analysis is shown in the right panel. **(D)** Efflux of Rh123 was measured using flow cytometry. **(E)** ABCB1, ABCC1 and Twist1 expression levels. The right panel shows the relative protein level. The expression levels of Twist1 and ABCB1/ABCC1 were upregulated in the HCT-8/V cell line compared with those in the HCT-8 parental cells. Twist1 silencing decreased the ABCB1 and ABCC1 expression levels in HCT-8/V cells. Data from three independent experiments are graphed as mean ± SD. *P < 0.05.

After the silencing of Twist1, the drug sensitivity was measured to confirm the critical role of Twist1 in the chemoresistance of HCT-8/V cells. As shown in Figure 6A, after Twist1 silencing, the RI values of vincristine, oxaliplatin, and taxol were 13.72, 26.26, and 15.32, respectively. These values suggested that the knocking down of Twist1 reversed the MDR of the colon cells. The decreased proliferation ability of the colon cells was observed after the Twist1 siRNA treatment in the colony-forming assays (Figure 6B). Increased apoptosis in the cells was also observed through the Annexin-PI assays (Figure 6C). Twist1 silencing increased the Rh123 accumulation in the tumor cells. Twist1 siRNA then enhanced the chemosensitivity of the drugs (Figure 6D). Moreover, Twist1 silencing decreased the ABCB1 and ABCC1 expression levels in HCT-8/V cells (Figure 6E). We also confirmed the above results of Twist1 mediated chemoresistance in Bel7402 hepatomacarcinoma cells (Supplementary Figure 5). These results further confirmed the regulatory effects of Twist1 on the ABCB1 and ABCC1 expression levels. Furthermore, a rescue experiment were performed and the results showed that the reversal effects of MDR by Twist1 silencing can be rescued by ABCB1 overexpression, confirming that ABCB1 is a function mediator of Twist-induced drug resistance (Supplementary Figure 6).

### Twist1 silencing enhanced the chemosensitivity of the HCT-8 xenograft to vincristine *in vivo*

**Figure 7:**
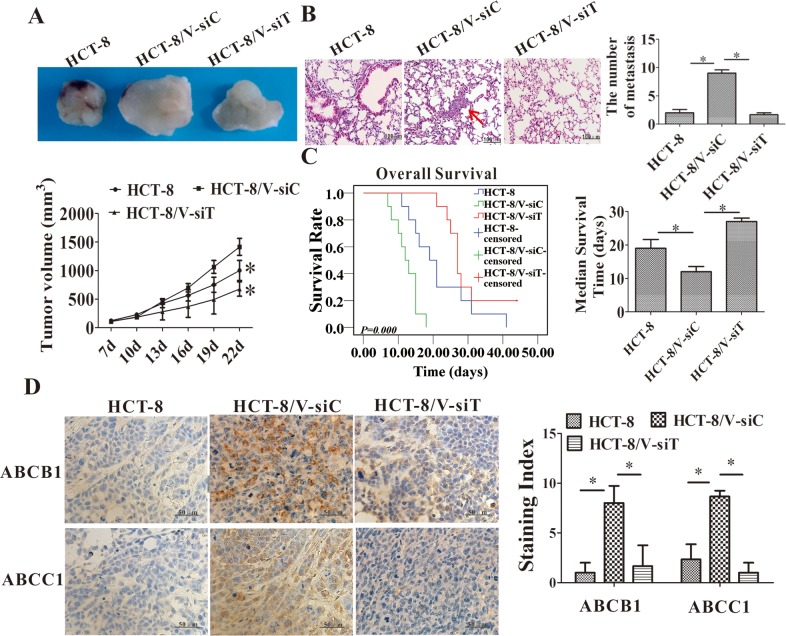
Downregulation of Twist1 increased the chemosensitivity of HCT-8 transplanted tumor to vincristine *in vivo* **(A)** Effects on the tumor growth rate. The left panel shows the representative images of tumors harvested at the end of the experiments; the right panel shows the curve of the xenograft growth. **(B)** Metastases in lung tissues of the tumor-bearing model. The left panel shows the representative images of lung tissues stained with H&E; the right panel shows the number of metastases in lung tissues. The scale bars represent 100 μm. **(C)** Overall survival analyses in mice treated with Twist1-siRNA or control siRNA followed by vincristine treatment (*P* = 0.000). **(D)** Immunohistochemical staining for ABCB1 and ABCC1 in tumors. Representative images are shown in the left panel; the staining index is shown in the right panel. Scale bars = 50 μm. Data from three independent experiments are graphed as mean ± SD. *P < 0.05.

HCT-8/V tumor-bearing mice were used to verify the drug reversal effects of Twist1 silencing. Twist1 siRNA treatment significantly inhibited the tumor growth and lung metastasis of the HCT-8/V xenograft (Figure 7A–7B). Moreover, the median survival time of HCT-8/V tumor-bearing mice was increased by 56% after the Twist1 siRNA treatment (Figure 7C). The ABCB1 and ABCC1 expression levels in the HCT-8/V xenograft tissues decreased after Twist1 silencing (Figure 7D). These results strongly suggested that Twist1 silencing enhanced the chemosensitivity of the HCT-8/V cells to vincristine.

## DISCUSSION

In the present study, we for the first time demonstrated that vincristine-resistant colon cancer exhibited changes in cellular morphology, motility, and molecular markers consistent with EMT. These results suggested a possible relatioship between vincristine-resistance and EMT induction in colon cancer.

EMT is closely related to the progression and metastasis of cancer [8]. Recent reports proposed that cell phenotype is responsible for their sensitivity to chemotherapeutic drugs, and epithelial-type cancer cells are highly susceptible to chemotherapeutic agents [9].

EMT regulators, such as Twist, Snail, Slug, and ZEB 1, are generally considered to play indispensable roles in EMT induction [10, 11]. Our results showed that Twist1 expression was upregulated in the HCT-8/V cells, and the ectopic expression of Twist1 induced MDR and increased ABCB1 and ABCC1 expression levels, as well as the drug-resistance-related genes participating in its signal pathway. Twist1 may promote MDR, possibly through the upregulation of ABCB1 and ABCC1.

siRNA targeting Twist1 was used to evaluate the effect of Twist1 on drug sensitivity and confirm the role of Twist1 in MDR. The siRNA-mediated suppression of Twist1 enhanced the drug sensitivity in the colon cancer cells. Thus, Twist1 silencing may be an important means to reverse EMT and restore the sensitivity of the HCT-8 vincristine-resistant cells.

ABC transporters were studied in this research because they have been proved as major players in cancer chemoresistance and leading to the progress of anticancer therapeutic strategies based on these transporters. ABCB1 is an energy-dependent drug efflux pump that exports chemotherapy drugs from the cells to the external environment [12]. Overexpression of ABCB1 decreases the intracellular accumulation of chemotherapeutic drugs, and this decrease subsequently induces MDR of the tumor cells to anticancer drugs [13]. Stein et al demonstrated that ABCB1 was overexpressed in clinical cancer with surgical samples of primary colon carcinomas [14]. ABCC1 can bind drug and glutathione separately or as a conjugate [15]. The expression levels of ABCB1 and ABCC1 are increased by many signaling pathways, such as the JNK pathway [16]. Our study demonstrated that Twist1 can induce MDR in colon carcinoma by promoting the expression of ABCB1 and ABCC1 *in vitro* and *in vivo*.

In summary, our data confirmed that Twist1 plays a vital role in MDR of colon carcinoma by upregulating ABCB1 and ABCC1. We also promoted a novel therapeutic strategy to overcome drug resistance through the inactivation of the Twist1 expression in colon cancer.

## MATERIALS AND METHODS

The entire sequence of the complementary DNA (cDNA) of Twist1 was generated using the total cDNA of normal human embryo. The latter was digested with XhoI/EcoRI, and subcloned into the plasmid pcDNA3.1 (Invitrogen, Carlsbad, CA). The resulting constructs were verified by DNA sequencing. Twist1 small interfering RNA (siRNA), Opti-MEM medium, control siRNA, and siRNA transfection reagent were purchased from Santa Cruz. Crystal violet, 3-(4,5-dimethylthiazol-2-y1)-2,5-diphenyltetrazolium bromide (MTT), and Rh123 were purchased from Sangon Biotech (Shanghai, China). Matrigel and transwell chambers were purchased from Biosciences (San Jose, CA, USA). ActinRed and Annexin V-FITC Apoptosis Detection Kit were purchased from KeyGEN BioTECH (Nanjing, China). Human recombinant TGF-β1 (rhTGFβ1) and anti-TGF-β1 neutralizing antibody was purchased from R&D Systems (Minneapolis, MN). The antibodies to ABCB1, ABCC1, vimentin, E-cadherin, and Glyceraldehyde 3-phosphate dehydrogenase (GAPDH) were purchased from Affinity Bioreagents (Colorado, USA). Annexin V-fluorescein isothiocyanate (FITC)-goat anti-mouse IgG and tetramethylrhodamine (TRITC)-goat anti-rabbit IgG were purchased from EarthOx (San Francisco, USA).

### Cell culture and transfection

The normal human colon mucosal epithelial cell line (NCM460), the low invasive human colon adenocarcinoma cell line (HT-29), the highly invasive human colon cancer cells (HCT-8), HCT-8/V and Bel7402 hepatocarcinoma cell line were purchased from KeyGEN BioTECH (Nanjing, China). All the cell lines were verified through short tandem repeat analysis. These cell lines were maintained in RPMI 1640 supplemented with 10% fetal bovine serum (FBS) (Hyclone, USA) in a humidified atmosphere of 5% CO_2_ at 37°C (Supplementary Table 1). The HCT-8/V cell line was preserved in 1.6 mg/L vincristine.

The cells (1×10^6^) were planted in a 60 mm dish. After the cells were incubated overnight, they were transfected with pcDNA3-Twist1, pcDNA3-negative, Twist1 siRNA, and control siRNA in serum-free Opti-MEM medium (Santa Cruz, California, USA) using Lipofectamine 2000 (Invitrogen, California, USA) according to the manufacturer’s instructions. The cells were collected after 48 h for the other experiments. To test for rescue of Twist 1 silencing, pcDNA3-ABCB1 vectors were co-transfected together with the Twist1 siRNA. For EMT induction, the cells were treated with 3 ng/mL of rh-TGF-β1 for 96 hours. The neutralizing TGF-β1 antibody was used to inhibit the TGF--β1 activity.

### Cell growth and chemosensitivity

The cells were seeded in 96-well plates at a density of 5×10^3^ cells per well and then cultured for 18 h before treatment with vincristine, taxol, and oxaliplatin. After 48 h, 100 μL of MTT was added to each well. The cells were then incubated for 4 h at 37°C. Finally, 150 μL of dimethyl sulfoxide was added to stop the reaction. The absorbance at 492 nm was measured, and the value of 50% inhibitory concentration (IC_50_) was determined. The resistance index (RI) was calculated by dividing the IC_50_ of the MDR cells by that of the parental sensitive cells. The reversal fold of MDR was calculated by dividing the IC_50_ of the cells with vincristine or other drugs in the absence of Twist1 siRNA by that obtained in its presence.

### RNA extraction and reverse transcriptase polymerase chain reaction (RT-PCR)

The total RNA was isolated using a TRIZOL reagent (Invitrogen Life Technologies), and cDNA was synthesized using a Gene-Amp RNA PCR cDNA synthesis kit (Applied Biosystems, Carlsbad, CA, USA). Polymerase chain reaction (PCR) was performed to amplify the specific cDNAs. Specific primers were designed using Primer 3 online tool. GAPDH were used as normalizing controls. The sequence of the primers used is provided in Supplementary Table 2.

### Cytoskeleton staining

The cells were seeded into the chamber slides at 1×10^5^ cells/well and cultured overnight. The coverslips were fixed with 3.7% formaldehyde for 10 min, washed with PBS, and treated with 0.1% Triton X-100 for 5 min, washed with PBS, stained with ActinRed in the dark for 20 min, washed with PBS, incubated with DAPI for 10 min at room temperature, washed with PBS, and finally observed using a confocal laser scanning microscope (Nikon, Japan).

### Efflux of Rh123

For the determination of the Rh123 efflux, the cells (5×10^5^) were loaded with 5 mg/mL Rh123 for 2 h in the dark. The medium was then replaced with Rh123-free medium. Following the efflux intervals of 1 h, the medium was removed. The cells were then washed with ice-cold PBS and placed in PBS containing 10% FBS on wet ice. The green fluorescence of Rh123 was detected by flow cytometer (easyCyte HT, Millipore, USA).

### Reporter gene assay

The ABCB1 and ABCC1 promoters were cloned into the pGL6-TA luciferase reporter vector (Supplementary Table 3). Transactivation assays were performed using the Dual-Luciferase Reporter Assay System (Mlbio, Shanghai, China) and measured using the Luminoskan Ascent Reader System (Thermo, Massachusetts, USA).

### Flow cytometric analysis of cell apoptosis

The cells were exposed to 0.8 μg/mL of vincristine for 48 h. The cells were then harvested by trypsinization and stained with FITC and propidium iodide (PI) (Annexin V-FITC Apoptosis Detection Kit) according to the manufacturer’s instructions. Subsequently, the apoptotic cells were analyzed by flow cytometry (easyCyte HT, Millipore, USA).

### Caspase-3/7 assay

Caspase-Glo® 3/7 Assay (Promega) was used to determine caspase-3/7 activity. The assay was used according to the manufacturers’ instructions. The HCT-8 cells (treated or untreated with TGF-β) were exposed to vincristine (80 nM) for 24 h. Caspase-Glo® 3/7 was added to the cells and incubated for 30 min. The caspase activity was determined in an LS55 Luminescence spectrometer (PerkinElmer, Waltham, MA, USA).

### Colony forming assay

A total of 500 cells were plated in six-well plates and cultured for 14 days. The cells were fixed with methanol and stained with 0.1% crystal violet solution. After washing and drying, the visible colonies were photographed through the microscope and then counted.

### Wound-healing assay

A total of 5×10^5^ live cells were seeded in the 6-well plates and grown to reach confluence. The cells were wounded with 200 μL tips and washed with PBS. After washing, the cells were incubated in a medium and observed under a microscope. Photographs were captured at 0, 6, and 24 h using a Nikon microscope. The distance of the migrated cells from the wound zone was measured using the following formula: healing rate = (distance before healing – distance after healing)/the distance before healing × 100%.

### Transwell assays

The transwell chambers were coated with Matrigel for 1 h at 37°C. The cells (2 × 10^5^) were added to the upper chamber containing 0.1% FBS medium to a total volume of 100 μL. The lower chamber contained 500 μL of RPMI 1640 supplemented with 10% FBS. After 24 h, the cells that migrated to the basal side of the membrane were fixed in methanol, stained with 0.1% crystal violet solution, photographed, and manually counted.

### Western blot analysis

The cells were harvested and lysed in a 50 μL cell lysis buffer containing protease inhibitors. The cell lysates were separated by 10% SDS-PAGE. The proteins were transferred to polyvinylidene fluoride membranes (Millipore, Billerica, MA, USA), blocked with TBST containing 5% skim milk powder, and then incubated at 4°C overnight with primary antibodies against ABCB1 (1:500), ABCC1 (1:500), E-cadherin (1:1500), vimentin (1:1500), cleaved PARP(1:500), Twist1 (1:1000), SNAIL (1:1000), and SLUG (1:100). The membranes were washed with TBST five times and then incubated with the appropriate HRP-conjugated secondary antibodies at room temperature for 1 h. The protein bands were detected by chemiluminescence.

### Immunofluorescent assays

The cells were seeded into the chamber slides at 1×10^5^ cells/well and then cultured overnight. The coverslips were fixed with 4% formaldehyde for 20 min, washed with PBS, treated with 5% FBS for 2 h at room temperature, and incubated with rabbit anti-human vimentin (1:100) or mouse anti-human E-cadherin (1:100) primary antibodies for 2 h. The cells were incubated with goat anti-mouse FITC-conjugated or goat anti-rabbit TRITC-conjugated secondary antibody for 1 h, incubated with DAPI for 10 min at room temperature, washed with PBS twice, and observed using a fluorescence confocal microscope.

### Chip-PCR analysis

The details of the Chip-PCR analysis are indicated in the Supplementary data.

### Microarray technology

The cells (1×10^6^) were planted in a 60 mm dish. After the cells were incubated overnight, the cells were transfected with pcDNA3-Twist1 and pcDNA3-negative using Lipofectamine 2000 according to the manufacturer’s instructions. After 48 h, the cells were collected for microarray analysis. Briefly, the total RNA was extracted and determinate concentration. Total Prep RNA Amplification Kit (Ambion, Austin, TX, USA) were used to amplification and biotin labeling. The Human HT-12, V4 expression BeadChip (illumine) were selected to perform microarray assay. The Gene expression model of Genome Studio software (V2011.1) were used to implement data analysis.

### Clinical sample analysis

The details of the clinical sample analysis are indicated in the Supplementary data.

### Tumor xenograft model and treatment

The HCT-8 cells (1×10^6^ per animal) or HCT-8/V cells (1×10^6^ per animal) were subcutaneously implanted into BALB/C nude female mice aged 4–6 weeks (n = 10 for HCT-8 and n = 20 for HCT-8/V). The mice were maintained under specific pathogen-free conditions. The animal procedures were performed according to the approved protocol by the Animal Ethical Committee of the Tianjin International Joint Academy of Biotechnology and Medicine. When the tumor volumes reached approximately 100 mm^3^, ten mice from the HCT-8/V group were randomly selected and administered with 0.5 mg/kg of Twist1-siRNA daily. All the groups were then provided with vincristine (2 mg/kg) treatment once every two days for two weeks. The tumor volume was measured regularly and calculated using the following formula: (*W^2^* × *L*)/2, where *W* represents the width, and *L* represents the length. The mice were sacrificed, and their tumor and lung tissues were harvested on day 22. The tissues were fixed in 4% formaldehyde, dehydrated, paraffin-embedded, and sectioned for hematoxylin–eosin (H&E) and immunohistochemistry (IHC) staining. Another 30 mice were allocated randomly into three groups as described above (10 mice per group) to measure the survival rates. Each mouse was injected with 1×10^7^ cells (suspended in PBS) into the caudal vein. The survival time of each mouse was recorded.

### Immunohistochemical analysis

The tissues were dehydrated in decreasing concentrations of ethanol and subsequently incubated with 3% hydrogen peroxide for 15 min to block endogenous peroxidase activity. The tissues were then treated with citrate buffer saline (pH 6.0) for 15 min at 95°C to retrieve the antigens. The tissues were then blocked by 10% FBS for 30 min at room temperature and incubated with different primary antibodies in a humidified chamber overnight at 4°C. 3,3′-diaminobenzidine and hematoxylin were used for color development and counterstaining, respectively. The primary antibodies and dilution ratios were listed as follows: rabbit polyclonal anti-Twist1 (1:200, Santa Cruz Biotechnology, California, USA), rabbit polyclonal anti-E-cadherin (1:100, Affinity, Cincinnati, USA), goat polyclonal anti-vimentin (1:50, Affinity, Cincinnati, USA), rabbit anti-ABCB1 (1:200, Sigma-Aldrich Chemical Co., St. Louis, Missouri, USA), and rabbit polyclonal anti-ABCC1 (1:50, Affinity, Cincinnati, USA). Negative controls were prepared using PBS instead of the first antibody.

The expression levels of E-cadherin, vimentin, ABCB1, and ABCC1 were independently evaluated by two investigators. The tumor cells with brown cytoplasm, nucleus, or membrane were considered positive. These cells were scored and then classified into the following four classes: none (0), weak brown (1+), moderate brown (2+), and strong brown (3+). The percentage of the stained tumor cells was divided into the following five classes: 0 for negative cells, 1 for 1%–25%, 2 for 25%–50%, 3 for 50%–75%, and 4 for >75%. The multiplication (staining index) of the intensity and percentage scores was used to determine the result. A staining index of ≥6 was defined as a high expression, whereas that of <6 was defined as a low expression [17].

### Statistical analysis

The differences among the groups were analyzed by one-way analysis of variance using Bonferroni post hoc test. Qualitative variables were analyzed using Fisher’s exact two tailed test or the chi-squared test. *P* < 0.05 was considered statistically significant. Data were analyzed using SPSS 17.0 (SPSS, Inc., Chicago, IL, USA) and presented as the mean ± standard deviation (SD).

## SUPPLEMENTARY MATERIALS FIGURES, TABLES AND REFERENCE







## Abbreviations

MDR: multidrug resistance
EMT: epithelial-mesenchymal transition
RI: resistance index
cDNA: complementary DNA
siRNA: small interfering RNA
IC50: 50% inhibitory concentration
FITC: annexin V-fluorescein isothiocyanate
PI: propidium iodide
GAPDH: glyceraldehyde-3-phosphate dehydrogenase
Rh123: rhodamine 123
IHC: immunohistochemistry
