# Supplementary material for: Twist1 confers multidrug resistance in colon cancer through upregulation of ATP-binding cassette transporters
Source: Oncotarget. 2017 May 2;8(32):52901–12. doi: 10.18632/oncotarget.17548 (PMC5581080; doi:10.18632/oncotarget.17548)
Supplement: Supplementary file 1 [file oncotarget-08-52901-s001.pdf]

Twist1 confer multidrug resistance in colon cancer through upregulation of ATP-binding cassette transporters

SUPPLEMENTARY MATERIALS

Chip PCR analysis

The HCT-8 cells transfected with pCDNA3.0-Flag-Twist1 were cultured for 72 h, then crosslinked with 1% formaldehyde for 10 min and quenched with 0.125 M glycine at room tempreture. Cells were lysed by adding lysis buffer, then sonicated to obtain chromatin fragments ranging between 300 and 700 bp. After precleared with protein-A agarose beads (Invitrogen, USA), the Twist1-bound chromatin was isolated using a rabbit anti-Flag antibody (Cell Signaling Technology, Beverly, MA, USA) or a rabbit anti-IgG antibody (isotype control) (Bangalore Genei) by incubation at 4°C overnight. 'No antibody' control was also incubated at 4°C. Complexes were washed with SDS buffer, and subjected to RNase (Sigma-Aldrich) and proteinase K (Sigma-Aldrich) treatment to reverse crosslinking at 65°C overnight. The ChIP DNA was purified by phenol–chloroform extraction. Primers specific were used to confirm Twist1 binding in E-box sites within the ABCB1 promoters. The sequences of the PCR primers used are

Fwd:TTTTCTCTCTGTGACAGCTCAGT;
Rev:AGCACAAATTGAAGGAAGGAGTAA.

Clinical sample analysis

We studied the expression of Twist1 and ABCB1 in paraffin embedded tissues of specimens from advanced colorectal carcinoma patients (stage III or IV). After obtaining informed consent in accordance with institutional guidelines, seventy tumor specimens from Shun Yi Hospital of Beijing were obtained. All patients were primarily treated with debulking surgery and postoperative vincristine chemotherapy.

During the period 2004-2013. Diagnosis was based on conventional morphologic examination of paraffin-embedded specimens and staging was based on pathological findings according to the American Joint Committee on Cancer classification.

REFERENCE

1. Boyd KE, Farnham PJ. Coexamination of site-specific transcription factor binding and promoter activity in living cells. Mol Cell Biol. 1999; 19: 8393-9.

SUPPLEMENTARY MATERIALS

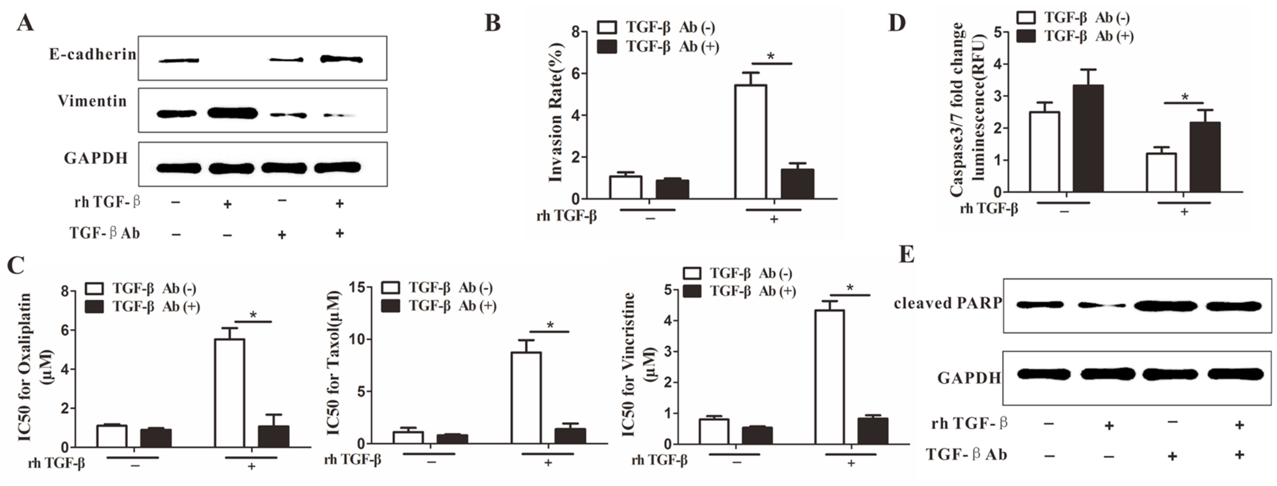

Supplementary Figure 1: TGF-β neutralizing antibody reversed EMT and MDR induced by rh TGF-β. **(A)** Western blot analysis showed that TGF-β neutralizing antibody decreased vimentin expression and increased expression of E-Cadherin. **(B)** TGF-β neutralizing antibody inhibits the invasion ability of rh TGF-β treated cells. **(C)** The drug sensitivity of the TGF-β-treated cells increased by addition of TGF-β neutralizing antibody. **(D)** and **(E)** TGF-β neutralizing antibody promoted caspase-3/7 activation and PARP cleavage. Data from three independent experiments are graphed as mean ± SD. *$P < 0.05$.

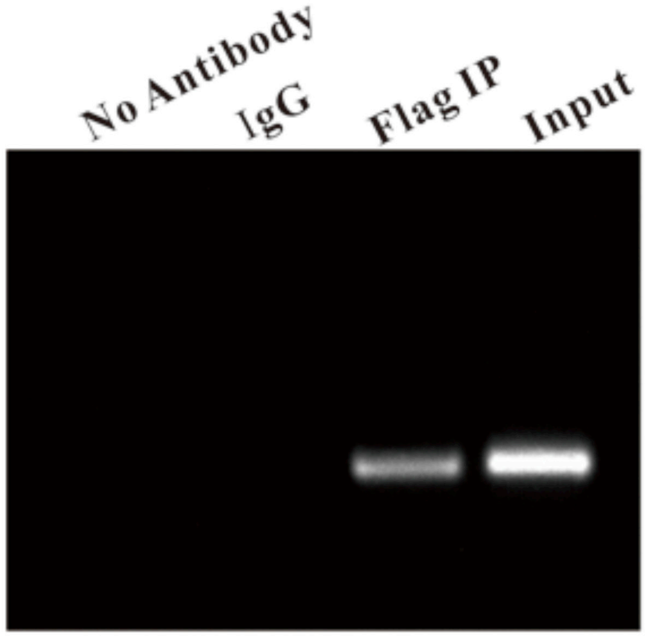

Supplementary Figure 2: Gel picture of PCR carried out on Chip undertaken in HCT-8 cells transiently overexpressing Flag-Twist using anti-Flag Ab. No antibody (No Ab) served as a negative control and rabbit IgG (Rab IgG) served as an isotype control.

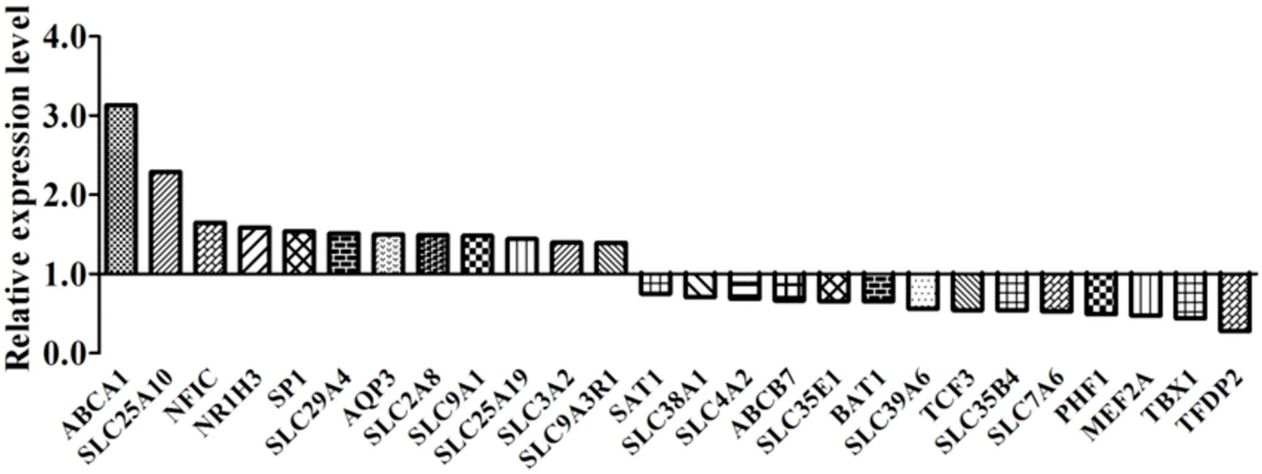

Supplementary Figure 3: Microarray technology was performed to evaluate the differentially expressed genes of cancer cells with Twist1 overexpressed.

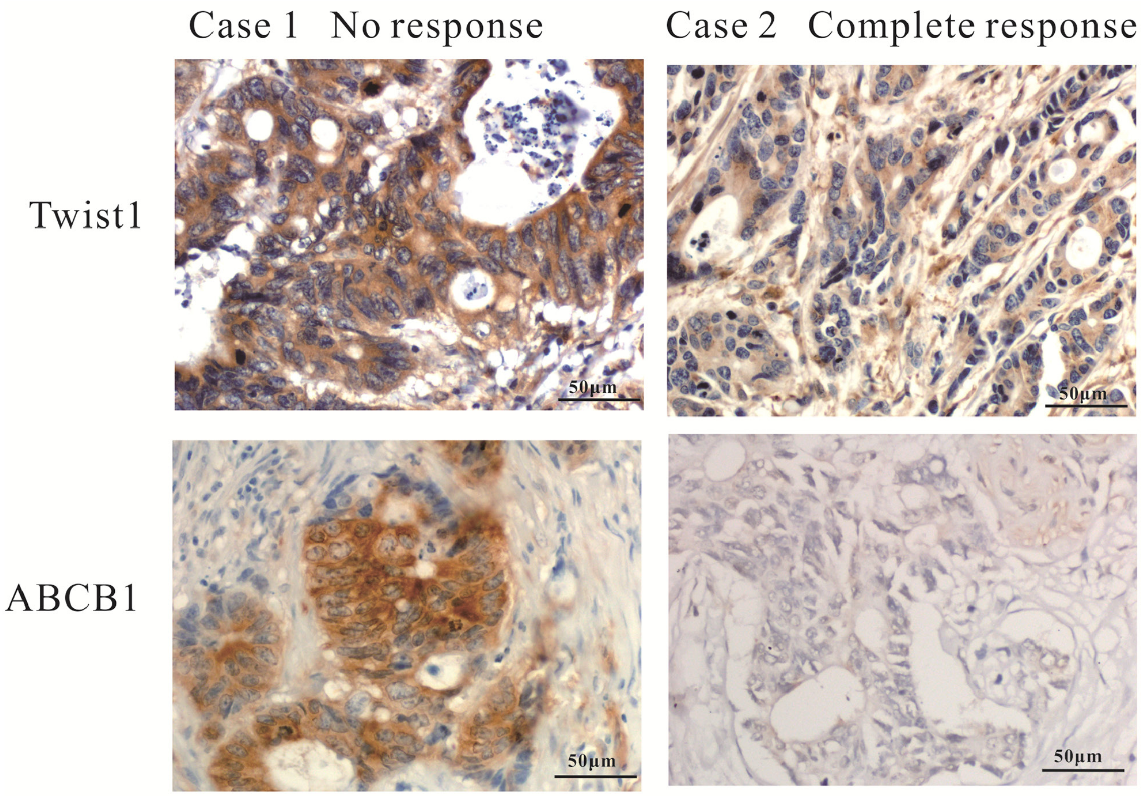

Supplementary Figure 4. Representative images of differential expression of Twist1 and ABCB1 in clinical colorectal cancer specimens with complete response vs no response. Scale bars = 100 μm.

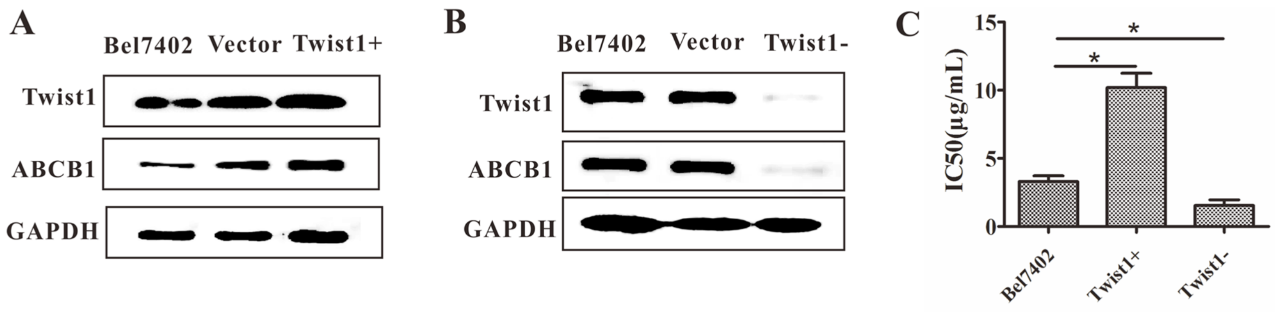

Supplementary Figure 5: Effects of Twist1 etopic expression or knockdown on the expression of ABCB1 and IC50 values to vincristine in Bel7402 cells. **(A)** Overexpression of Twist1 resulted in increased expression of ABCB1. **(B)** Knockdown of Twist1 resulted in decreased expression of ABCB1. **(C)** Effects of different Twist1 expression on the IC50 values to 5-FU. Data from three independent experiments are graphed as mean ± SD. *P < 0.05.

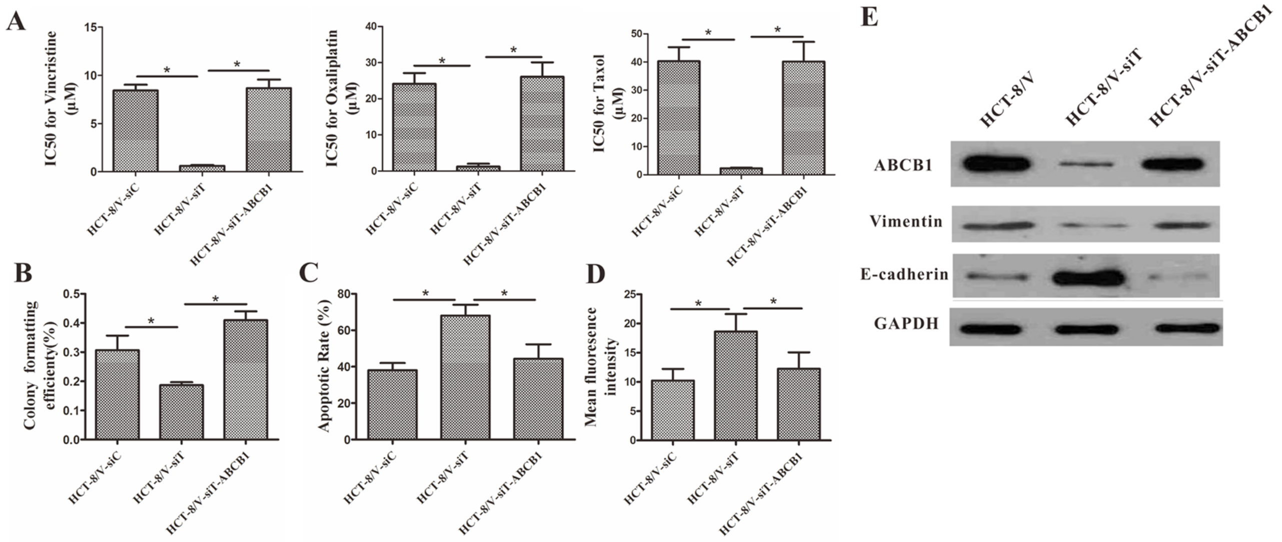

Supplementary Figure 6: The reversal effects of MDR by Twist1 silencing can be rescued by ABCB1 overexpression. **(A)** ABCB1 overexpression restored the IC50 values in HCT-8/V cells with Twist1 silencing. **(B)** Colony formation assays, (C) apoptosis assays and (D) Rh123 efflux analysis by flow cytometry. All reflect the rescuce effects of ABCB1 overexpression on MDR reversion by Twist1 silencing. **(E)** ABCB1 overexpression restored the mesenchymal phenotype in HCT-8/V cells with Twist1 silencing. Data from three independent experiments are graphed as mean ± SD. *P < 0.05.

Supplementary Table 1: Overview of the cell lines.

See **Supplementary File 1**

Supplementary Table 2: The sequences of the primers used in reverse transcriptase polymerase chain reaction.

See **Supplementary File 2**

Supplementary Table 3: ABCB1 and ABCC1 promoter reporter clones

Promoter reporter clones	Promoter sequence	Vector information
ABCB1	Promoter Length: 1269 bp	pEZX-PG04
	Sequence length upstream of TSS: 949 bp	
	Sequence length downstream of TSS: 320 bp	
ABCC1	Promoter Length: 1582 bp	pEZX-PG04
	Sequence length upstream of TSS: 1407 bp	
	Sequence length downstream of TSS: 175 bp	

The promoter reporter clones were purchased from GeneCopoeia (Guangzhou, China)

Supplementary Table 4: Relationship between Twist1/ABCB1 expression and response to cancer therapy

Variant	No. of patients	No. of patients (%)			*P*
		Complete response	Partial response	No response	
Twist1(-)/ABCB1(-)	16	12	2	2	.000* .000#
Twist1(+)/ABCB1(-)	6	1	2	3	
Twist1(-)/ABCB1(+)	8	0	2	6	
Twist1(+)/ABCB1(+)	40	1	6	33	

(-) low expression; (+) high expression; * by chi-square test; # by Fisher test.
