# Supplementary material for: Twist1 confers multidrug resistance in colon cancer through upregulation of ATP-binding cassette transporters
Source: Oncotarget. 2017 May 2;8(32):52901–12. doi: 10.18632/oncotarget.17548 (PMC5581080; doi:10.18632/oncotarget.17548)
Supplement: Supplementary file 2 [file oncotarget-08-52901-s002.doc]

**Supplementary Table 1: Overview of the cell lines**

| **Cell lines** | **Source** | **Growth properties** | **Morphology** | **Propagation** | | | **Subculturing** | **Preservation** | |
| --- | --- | --- | --- | --- | --- | --- | --- | --- | --- |
|  |  |  |  | **Medium** | **T** | **Atmosphere** |  | **Medium** | **T** |
| HCT-8 | Colon cancer | Adherent | Epithelial | RPMI1640+10%FBS | 37℃ | 95% air  5% CO_2_ | 1、remove medium  2、rinse with 0.25% trypsin, 0.03% EDTA solution  3、remove the solution and add an additional 1 to 2ml of trypsin-EDTA solution  4、allow the flask to sit at room temperature until the cells detach  5、add fresh culture medium, aspirate and dispense into new culture flasks  6、Subcultivation ratio: 1:3 to 1:5  7、Medium renewal: 2 to 3 times per week | Complete growth medium supplemented with 10% DMSO | liquid nitrogen vapor phase |
| HCT-8/V | Colon cancer | Adherent | Epithelial | DMEM (high glucose)+10%FBS |  |  |  |  |  |
| Bel7402 | Hepatocellular carcinoma | Adherent | Epithelial | DMEM (high glucose)+10%FBS |  |  |  |  |  |
