# Supplementary material for: Twist1 confers multidrug resistance in colon cancer through upregulation of ATP-binding cassette transporters
Source: Oncotarget. 2017 May 2;8(32):52901–12. doi: 10.18632/oncotarget.17548 (PMC5581080; doi:10.18632/oncotarget.17548)
Supplement: Supplementary file 3 [file oncotarget-08-52901-s003.doc]

**Supplementary Table 2: The sequences of the primers used in reverse transcriptase polymerase chain reaction**

| Gene name | Primer sequence (5'→ 3') |
| --- | --- |
| ABCA2 Fwd | GCCTCATTCCTCCAGTTGTT |
| ABCA2 Rev | CGAAACCAGAGGCTGAGGAT |
| ABCA3 Fwd | AGGAAAGGAGGCTGAAGGAG |
| ABCA3 Rev | GGTGCTGACCATGAAGCTGAAG |
| ABCB1 Fwd | GTTGGAGGTGAGATTAATTTT |
| ABCB1 Rev | AAACCCCCAACTCTACCT |
| ABCB4 Fwd | GCATCAGCAGCAAACAAAAA |
| ABCB4 Rev | GCAGCGACAAGGAAAAGTTC |
| ABCB5 Fwd | TTCATCCTCCGTGGCTTATC |
| ABCB5 Rev | ACGATTGCTATTTGGGAACG |
| ABCB11 Fwd | AGCAGCCTTTCTTCAGGTGAC |
| ABCB11 Rev | TCACCAGACATTCTACCAACAAC |
| ABCC1 Fwd | TTTATAGGATGAAATGAGGGTATAGT |
| ABCC1 Rev | AACAACCCA ACCAACCACCTCT |
| ABCC2 Fwd | CTTCGGAAATCCAAGATCCTGG |
| ABCC2 Rev | TAGAATTTTGTGCTGTTCACATTCT |
| ABCC3 Fwd | GGACCCTGCGCATGAACCTG |
| ABCC3 Rev | AGGCAAGTCCAGCATCTCTGG |
| ABCC4 Fwd | GGCAGTGACGCTGTATGG |
| ABCC4 Rev | CGCCAGGTCTGACAGTAAAG |
| ABCC5 Fwd | CACCATCCACGCCTACAATAAA |
| ABCC5 Rev | CACCGCATCGGCACACGTA |
| ABCC6 Fwd | CACTGCGCTCCAGGATCAGC |
| ABCC6 Rev | CAGACCAGGCCTGACTCCTG |
| ABCC10 Fwd | AAAATAAGCATACCCTATAGCATAA |
| ABCC10 Rev | AAAGGAAGATCATCATCAATAGAAT |
| ABCC11 Fwd | GGCTGAGCTACTGGTTGGAG |
| ABCC11 Rev | GCGATGAGCAACTGAAATGA |
| ABCC12 Fwd | GGTGTTCATGCTGGTGTTTGG |
| ABCC12 Rev | GCTCGTCCATATCCTTGGAA |
| ABCG2 Fwd | GTTTGATTTAGTTGGGTTTGG |
| ABCG2 Rev | AACCACCC ATTTAACTTACTCT |
| Twist1 Fwd | GCAAGAAGTCGAGCGAAGAT |
| Twist1 Rev | GCTCTGCAGCTCCTCGAA |
